# Kisspeptin and Cancer: Molecular Interaction, Biological Functions, and Future Perspectives

**DOI:** 10.3389/fendo.2018.00115

**Published:** 2018-03-27

**Authors:** Vincenza Ciaramella, Carminia Maria Della Corte, Fortunato Ciardiello, Floriana Morgillo

**Affiliations:** Medical Oncology, University of Campania “Luigi Vanvitelli”, Naples, Italy

**Keywords:** kisspeptin, metastasis, cancer, GPR54, prognostic biomarkers

## Abstract

Cancer disease is the second leading cause of death in the world and one of the main fields of medical research. Although there is now a greater understanding of biological mechanisms of uncontrolled cell growth, invasiveness and metastasization, the multi-step process of cancer development and evolution is still incompletely understood. The inhibition of molecules activated in cancer metastasization is an hot topic in cancer research. Among the known antimetastatic genes, KiSS-1 is involved in the metastatic cascade by preventing growth of metastasis. Moreover, loss of KiSS-1 protein expression by tumor cells has been associated with a more aggressive phenotype. *KiSS-1* gene encodes a 145-amino acid protein, which following proteolytic cleavage, generates a family of kisspeptins (Kp-10, -13, and -14), that are endogenous agonists for the G-protein-coupled receptor (GPR54). The antitumor effect of KiSS-1 was primarily associated with the inhibition of proliferation, migration and cell invasion and, consequently, the reduced formation of metastasis and intratumoral microvessels. In this review, we highlight the latest data on the role of kisspeptin signaling in the suppression of metastasis in various cancer types and the use modulators of KiSS/GPR54 signaling as potential novel therapeutic agents for the treatment of cancer.

## Introduction

Cancer is defined as a heterogeneous group of diseases, characterized by uncontrolled cell growth.

Currently, cancer disease is still one of the main causes of death, 7.6 million cancer deaths occurred in 2008 and it is expected that cancer related mortality could reach 13.1 million deaths by 2030 (1). The cancer mortality rate is caused in 90% of cases but the development of metastasis, that are the clinical result of the extremely invasive behavior of cancer.

Metastasization is the process through which primary tumor cells colonize distant second sites: a tumor cell that grows in a microenvironment, in order to become able to move and proliferate in another location, begins a chain of events defined as a “metastatic waterfall.” In particular, uncontrolled cell growth and tumor progression are the result of a multi-step complex process including inactivation of tumor suppressor genes. Tumor cells are characterized by epithelial to mesenchymal transition (EMT), a complex biological and morphological change that reduce their dependency from intracellular connection. Thus, mesenchymal tumor cells are able to infiltrate distant organs escaping the recognition from the immune system, until they create a macroscopic mass in second sites (2).

In the recent years, oncology research has focused its interest in identifying the metastases suppressor genes. At present, about 30 metastasis suppression genes have been isolated which provide useful candidates for cancer specific therapeutic strategies involving gene transfer, gene expression induction, exogenous administration of a genetic product, targeting of metastasis suppressors, and signaling (3).

**Figure 1:**
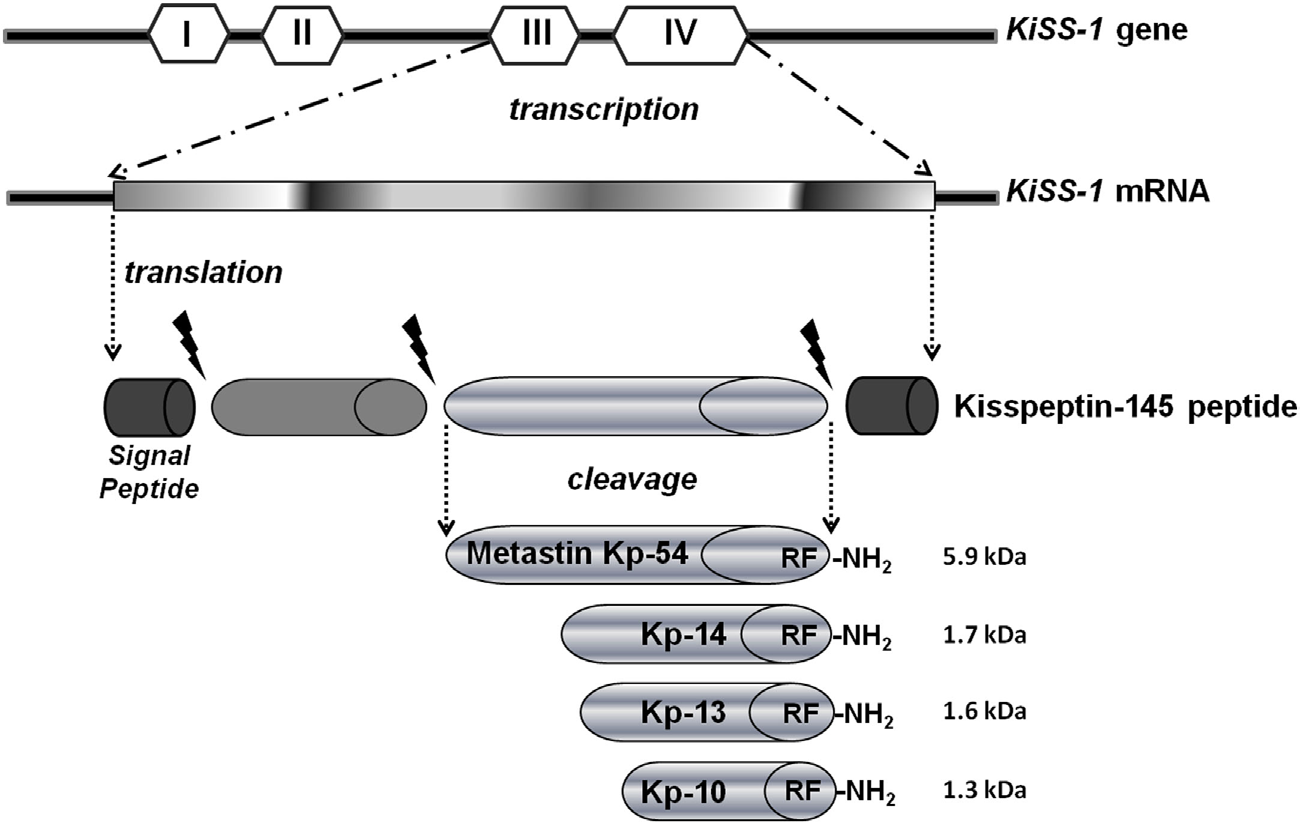
Kisspeptins (Kps) formation.

Among the known metastasis suppressor molecules, in this review, we highlight the KiSS-1 data suggests that its unique mechanism of action is capable of delaying the metastatic cascade by preventing growth and colonization of metastatic cells in distant sites. This mechanism is different from other antimetastatic genes, which block cellular detachment and migration from the primary tumor (4). The *KiSS-1* gene is located in the long arm of human chromosome six and encodes a 145-amino acid protein, which is subsequently cut to generate a C-terminal amidated 54-amino acid peptide kisspeptin-54 (Kp-54, called metastin after its capability to blockade metastasis), that is further cleaved into even shorter peptides (Kp-10, -13, and -14 amino acids long), generally defined as kisspeptins (Kps). All C-terminal cleavage fragments Kp-54, -14, -13, and -10 possess biological activity and are the endogenous ligands for the G-protein-coupled receptor (GPR54) (5) (Figure 1). Furthermore, it is demonstrated that treatment with KiSS-1 derived polypeptides reduced metastasis in mice engrafted with melanoma cells with GPR54 over-expression (6).

In this review, we present findings from preclinical and clinical settings and confer strategies, whereby KiSS-1 and its receptor GPR54 mediated signaling pathway which may be exploited as anticancer therapy in metastatic cancers.

## Kisspeptin in Cancer Proliferation and Metastasization

The antimetastatic action of the Kp was observed for the first time in human melanoma cell lines. The experiments based on hybridization and differential display showed the presence of an unregulated gene in cells transfected with chromosome six and this gene acted as a suppressor of metastasis; in fact, mapping demonstrated that this gene was not localized on chromosome six but on chromosome 1q32, and it was the *KiSS-1* gene (7, 8). Activation of the Kps/GPR54 system has been demonstrated to have a multiplicity of effects on cancer cell biology, including suppression of motility, culture scratch repair, proliferation, metastasis, and invasion of human cells *in vitro*. Until now, the specific mechanism for the antimetastatic function of Kps is still uncertain but various intracellular signaling pathways triggered by Kp have been identified that might be involved (9).

GPR54 belongs to the group A G-protein-coupled receptors that are associated with Gq/11, determining activation of the phospholipase C (PLC) signaling pathway and subsequently Ca_2_ recruitment. Stimulation of GPR54 by Kps also leads to phosphorylation of focal adhesion kinase (FAK) causing the formation of excessive focal adhesions and stress fibers (5), thus, explaining the action of Kps on inhibiting chemotaxis. Moreover, GPR54 activation reduces calcineurin activity (10), which contributes to the metastasis suppression. Until now, the antimetastatic activity of Kps has been demonstrated in several tumors including melanoma (6), thyroid, ovary, bladder, gastric, pancreas, and lung cancers (10–15). For some of these tumors, a unified antimetastatic mechanism regulated by the Kps/GPR54 system is related to the inhibition of the activity of the matrix metallo proteinase 9 (MMP-9) and the consequent blockade of tumor cell migration and invasion (16).

A molecular mechanism of Kp action is an inhibition of cellular proliferation during intracellular Ca^++^ replaces discharge with release and activation of protein kinase C (17). A possible mechanism of Kp action is through an increase in intracellular Ca^++^ which inhibits cell proliferation and increases cell differentiation and apoptosis. Thus, *KiSS-1* gene acts as a cancer suppressor gene and activation of Kp signaling cascade inhibits cancer cell invasion, metastasis formation, and tumor recurrence (13).

**Figure 2:**
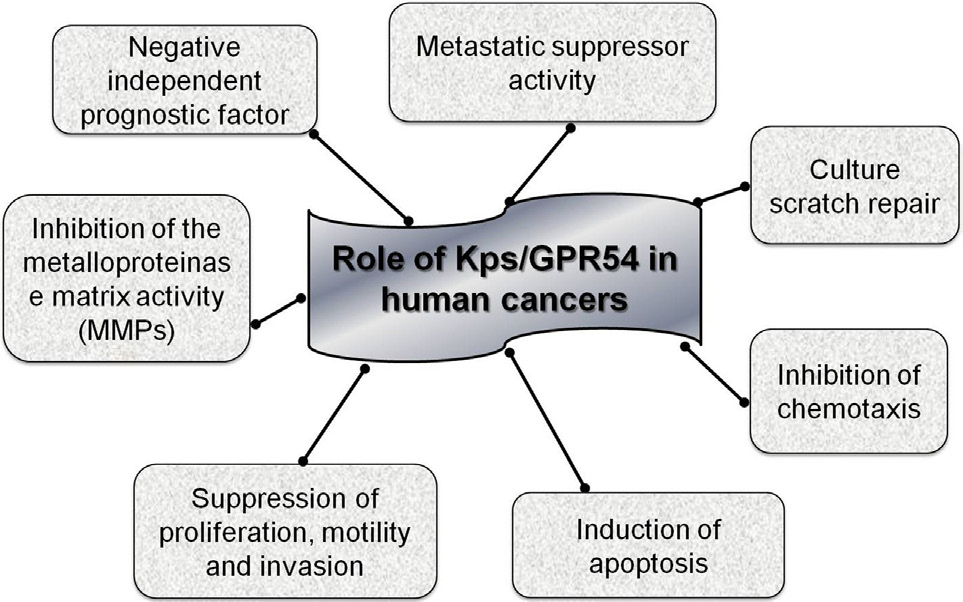
Kps/GPR54 in human cancer.

Contradictory information on the function of KiSS-1 and GPR54 in carcinogenesis may derive from the presence of alternative forms of these genes or their different epigenetic regulation. There is currently no confirmed data on the methylation status of *KiSS-1* and *GPR54* genes in cancer, but the study of this epigenetic modification could potentially elucidate the variations in expression patterns examined in different studies on human cancer tissues, as subsequently described (Figure 2).

## Expression of KiSS-1 and GPR54 in Human Cancers

According to various clinical data, a decrease in KiSS-1 and/or GPR54 expression has been shown to be associated with poorer prognosis in cancer patients and, therefore, the expression of KiSS-1 and/or GPR54 might be useful predictive biomarkers in medical outcomes.

From a case study on gastric patients, classified as low or high KiSS-1 expression levels, a down regulation of KiSS-1 was associated with recurrent cancer invasion and linked to shorter survival in several studies, suggesting that KiSS-1 could develop into a novel negative predictive factor for gastric cancer (13).

Similarly, in ovarian cancer patients, lower *KiSS-1* gene expression was related to more resistant ovarian cancers, cell invasion, the presence of macroscopic residual tumor following surgical resection, and the patient’s worse prognosis (11).

Similar data were detected in an analysis of urinary bladder cancer samples: loss of KiSS-1 expression was found in all invasive cancers and the surrounding normal uroepithelium showed higher levels of KiSS-1 expression. Moreover, KiSS-1 expression was also shown to be negatively correlated with the histopathological stage, with lower expression of the *KiSS-1* gene observed in cases with positive vascular invasions (12), confirming that KiSS-1 expression having a predictive value and correlating with negative prognostic features and worse patients’ outcome. In esophageal squamous cell carcinoma, lower KiSS-1expression is found in more of 85% of tumors with lymph nodes metastases, without any correlation with local invasion or primary tumor size, reinforcing the idea that Kp signaling plays a role in metastatic diffusion of cancer also in esophageal carcinoma (18).

A role of Kps has also been shown in breast cancer although the results are somewhat contradictory. Some chapters show that KiSS-1 mRNA and protein expression are deficient in node-positive cancers, and establish a considerable negative association with axillary lymph node attachment (19). In contrast, the results obtained by another study of KiSS-1 expression in high-risk breast cancer patients on surgical samples support the hypothesis that the *KiSS-1* gene is a metastasis suppression gene. In fact, it has been observed that the KiSS-1 transcriptional activity is present in node-negative breast cancer patients, primary breast cancer, and metastatic deposits, but higher expression, due to the molecular alteration, occurs essentially in localized tumors with no nodal involvement, thus confirming that restoring the function of the *KiSS-1* gene could be a promising approach to stop micrometastatic growth and prevent metastatic diffusion in distant sites (20). In other reported cases, the level of KiSS-1 mRNA and protein was higher in primary localized breast tumors than in breast cancer that was metastasized to other sites such as in the brain.

In contrast, a reduction of KiSS-1 expression in brain metastases has observed, thus suggesting that a loss of KiSS-1 may influence the formation of distant metastases; we speculate that further investigations on KiSS-1 expression levels in metastastic lesions and primary tumors in breast cancer patients could confirm the antimetastatic role of Kp (21).

No clinical data are available with regard to the correlation between KiSS-1 and GPR54 expression levels and the resistance to treatment. Only in metastatic prostate cancer patients, a preliminary data that increased expression of KiSS-1 is able to sensitize cancer cells to chemotherapies (22, 23); in the same setting of patients, expression of KiSS-1 is inversely correlated with tumor differentiation and clinical staging.

## Promising Role of Kisspeptin in Cancer Therapeutics

Several reports have attempted to demonstrate whether the secretion of Kps is necessary for metastasis inhibition. It has recently been shown that in cell lines, after transfection and blockade generation of metastases, the expression of the *KiSS-1* gene induces the production of the GPR54 receptor.

This autocrine feedback signal could represent a mechanism of perpetuation of antimetastatic effect of Kp (24).

The primary cancer cell lines derived from skin and lung had different levels of GPR54 expression, particularly, it has been demonstrated that lung cancer cells produce more growth inhibitory signals than skin cell cancer from primary cultures. These results confirm why melanoma cells expressing KiSS-1 are able to grow in the skin, but fail to develop after they have already distributed (25, 26).

Since the active peptides of KiSS-1 are secreted proteins, their effects can be simulated experimentally by delivery to the bloodstream. The idea that these molecules, Kps and synthetic derivatives, can be used in clinical setting as drug is very promising because as they are natural peptides, they should not generate serious toxic effects in humans (27). For example, their application could be compared to the use of insulin in diabetic patients.

A therapeutic option for use of Kps as treatments in humans could be tested if their injection in the human body could reach a systemic distribution and access tumor cells. The administration of Kps for therapeutic purposes has already verified as safe in humans: in fact, a single dose of Kp-54 administered subcutaneously did not cause any significant adverse effects. However, regular administration of this dose could affect the normal endocrine function and beginning of puberty due to the activation of the release of gonodotropin-releasing hormone (GnRH) (28). KiSS-1-based treatments could be hypothetically simple if metastatic tumor cells express the KiSS-1 receptor; but the limitation is that most tumor cells do not express GPR54, so it could be a challenging aim for future researches to test multiple cancer treatment with *KiSS-1* gene in the absence of its potential biomarker GPR54 (24).

Collectively, available preclinical data suggested that induction of *KiSS-1* expression gene and the therapeutic use of Kps could block metastasization. In particular, the current hypothesis is that KiSS-1 expression alone might have the ability to inhibit metastatic growth in multiple organs, by targeting disseminated cells and their interactions with the microenvironment, thus deserving future studies for Kps as novel potential anticancer molecular agents.

## Author Contributions

VC and CD wrote the chapter and made bibliography research. FC and FM revised the manuscript.

## Conflict of Interest Statement

The authors declare that the research was conducted in the absence of any commercial or financial relationships that could be construed as a potential conflict of interest.
